# Effects of Using a Smartphone App Combined With Behavior Change Techniques on the Level of Physical Activity Among Adults and Older Adults: Sequential Multiple Assignment Randomized Trial

**DOI:** 10.2196/73388

**Published:** 2026-01-30

**Authors:** Maria do Socorro Morais Pereira Simoes, Neli Leite Proença, Vinícius Tonon Lauria, Matheus Bibian do Nascimento, Ricardo da Costa Padovani, Victor Zuniga Dourado

**Affiliations:** 1 Departamento de Ciências do Movimento Humano, Instituto de Saúde e Sociedade, Universidade Federal de São Paulo, 136 Silva Jardim St, Room 338, Santos, 11.015-020, Brazil, 55 13-99800-0315; 2 Departamento de Saúde, Educação e Sociedade, Instituto de Saúde e Sociedade, Universidade Federal de São Paulo, Santos, Brazil; 3 Department of Global Health and Population, Bernard Lown Scholars in Cardiovascular Health Program, Harvard T.H. Chan School of Public Health, Harvard University, Boston, MA, United States

**Keywords:** physical activity, adaptive clinical trial, transtheoretical model of behavior change, smartphone, app

## Abstract

**Background:**

The use of tools, such as smartphone apps, to increase the level of physical activity (PA) decreases over time. Adaptive intervention trials have been recommended to test technology-based interventions owing to the possibility of adapting interventions based on individual responses.

**Objective:**

This study aimed to investigate the effects of using a smartphone app combined with behavior change techniques on the PA level in adults and older adults (assessed using the step count). Moreover, the study investigated the time spent in sedentary behavior and time spent in moderate-to-vigorous PA (MVPA).

**Methods:**

In this single-blinded, sequential multiple assignment randomized trial, participants were randomized into 3 groups during a 24-week intervention (group 1: app with tailored messages; group 2: app with tailored messages plus gamification I; and control group: educational information). In the sixth week, participants from groups 1 and 2 were classified as responders and nonresponders according to their average daily step count. Nonresponders were rerandomized among the other groups, adding a second type of gamification (group 3: app with tailored messages plus gamification II). After another 6 weeks, participants were reassessed and advised to keep monitoring their step count with the app, but without interference from the researchers. Face-to-face assessments were conducted. The behavior change techniques included app features (goal setting, auto-monitoring, ranking, and virtual badges) and researcher-provided resources (tailored messages and in-person sessions of PA). The intervention effects were analyzed using linear mixed models.

**Results:**

The study included 53 participants (control group: n=17, group 1: n=17, group 2: n=19; mean age 44.0, SD 12.7 years). Groups 1 and 2 had 63% (10/16) and 47% (7/15) responders, respectively (*P*=.38). Regarding the PA level, participants from group 1 showed increases in the average daily step count at all assessments (final vs initial: B=797.2 steps/day, 95% CI 475.3-1119.1; *P*<.001; follow-up vs initial: B=2097.6 steps/day, 95% CI 1577.2-2618.1; *P*<.001). All participants showed a reduction in the time spent in sedentary behavior at the final assessment compared with the initial assessment (B=−70.8 min/week, 95% CI −88.8 to −52.9; *P*<.001), without differences among groups. The time spent in MVPA varied across time among all participants. Regardless of the initial group and allocation in the second randomization, responders from groups 1 and 2 showed a constant increase in the average daily step count (week 6 vs week 1: B=1548.0 steps/day, 95% CI 1407.4-1688.6; *P*<.001; week 12 vs week 1: B=1720.3 steps/day, 95% CI 1568.8-1871.7; *P*<.001; week 12 vs week 6: B=172.3, 95% CI 20.8-323.8; *P*=.03).

**Conclusions:**

The adaptive intervention protocol using a smartphone app with behavior change techniques increased participants’ PA levels. Stepping up behavior change techniques and progressively offering new stimuli may contribute to a change in behavior regarding PA.

## Introduction

Despite the well-known benefits of physical activity (PA) in the prevention and treatment of several health conditions [1,2], a significant proportion of the global population does not meet its minimal recommended level [3]. The World Health Organization aims to reduce physical inactivity by 15% by 2030 [3]. Recent studies emphasize the complex interaction of individual [1,4,5], social [1,4,5], environmental [1,4,5], and political [4,5] factors influencing the practice of PA, suggesting that interventions should consider them [4,5]. Specifically, regarding individual and social factors, popular technologies, such as mobile devices and apps, have been used to increase the engagement of insufficiently active individuals.

Using smartphones as part of intervention programs can facilitate the auto-monitoring of PA, contributing to the behavior change process [6,7]. Duncan et al [6] compared auto-monitoring interventions for PA and eating habits using a website and printed material. They showed that both delivery methods improved the behavior, with no differences between groups. Evidence with moderate effects suggests that app-based interventions and pedometers may be effective [8]. Still, the association between app use and behavior change techniques may be more effective than app-based interventions alone [7].

Among the most commonly used behavior change techniques to promote PA are tailored messages, health education, gamification (the use of game elements in contexts other than games), and social support [7]. Regarding apps for PA and health, few are based on behavior change techniques. Among at least 25 behavior change techniques, only 1-8 techniques are offered by apps [9-12].

Despite the promising use of technologies, such as websites and smartphone apps, to deliver interventions targeted to increase the level of PA, studies show that the use of such devices decreases over time [6,7]. Interestingly, behavior change usually presents with the same pattern: individuals begin the program more engaged and active but do not maintain the new behavior for a long time [13]. That said, adaptive intervention trials, such as the sequential multiple assignment randomized trial (SMART), have been recommended to test technology-based interventions instead of classical clinical trials [14,15] due to the possibility of adapting interventions over time based on individuals’ responses. It would be reasonable to extrapolate this rationale to interventions targeted to promote PA. Clinical trials with a SMART design have been shown to be feasible [16,17], but so far, this design has been barely used for PA interventions [17].

Our primary aim was to investigate the effects of using a smartphone app combined with behavior change techniques on the level of PA among adults and older adults, assessed by the average number of daily steps. Our secondary aim was to investigate the effects of the intervention on time spent in sedentary behavior and time spent in moderate-to-vigorous PA (MVPA), assessed by a triaxial accelerometer.

We hypothesized that the smartphone app combined with behavior change techniques offered in a SMART adaptive intervention design would be an effective tool to increase the level of PA among adults and older adults.

## Methods

### Study Design

This study had a single-blinded SMART design, in which the assessor was blinded regarding group allocations, with a 1:1 allocation ratio. The protocol was registered at the Brazilian Register of Clinical Trials (RBR-8xtc9c), and the study has been presented according to the CONSORT (Consolidated Standards of Reporting Trials) 2025 [18] and CONSORT-EHEALTH (V.1.6.1) [19] recommendations (Checklist 1). We enrolled participants continuously from November 2018 to February 2020. The study protocol was previously published in detail [20].

The primary outcome of the study was the average number of daily steps, and the secondary outcomes were the time spent in sedentary behavior and the time spent in MVPA, which were all assessed by a triaxial accelerometer.

### Ethical Considerations

The project was approved by the Local Ethics Committee of the Federal University of São Paulo (CAAE 89112418.8.0000.5505), and all volunteers signed an informed consent form in person before participation. The informed consent form contained detailed information about the assessments and the group allocation, including a detailed description of the control and intervention groups. Participants did not receive any compensation before, during, or after the study. Only the principal researchers had access to participants’ personal information. No identifiable information and/or images were or will be published.

### Participants and Setting

We recruited participants through social media posts, distribution of printed material in different neighborhoods, and recommendations from other participants. The content of the recruitment material included the question “Do you want to increase your level of physical activity? We invite volunteers aged from 20 years, users of smartphones, that desire to move more. During 6 months, we will follow you to promote physical activity. Contact us!” or “Do you consider yourself a not very active person? Do you want to increase your level of physical activity? The EPIMOV Laboratory is looking for volunteers aged 20 years and older, able to walk without assistance from another person, and free of cardiac or pulmonary diseases. Contact us!” Those interested in participating were required to call or text a number to schedule the assessment. Participants did not receive any type of incentive to cooperate in the study.

The inclusion criteria were as follows: (1) age 20 years or older; (2) absence of diagnosed cardiopulmonary, locomotor, or other conditions that could preclude the safe unsupervised performance of PA; and (3) having a smartphone and being familiar with its use, which was assessed by researcher observation and judgment during the enrollment phase.

The exclusion criteria were as follows: (1) basal level of PA of ≥10,000 average steps per day; (2) self-reported recent respiratory infection; (3) abnormalities on the cardiorespiratory fitness test that preclude the safe performance of unsupervised PA; and (4) refusal to participate by signing the informed consent. Although Tudor-Lock et al [21] suggested that interventions to increase the level of PA should mainly target individuals who perform less than 5000 steps per day, we decided not to restrict the sample to sedentary individuals and set the limit of 10,000 steps per day, which indicates a considerable amount of PA. This criterion was assessed using a triaxial accelerometer, worn by participants for 7 consecutive days. The procedure is described in detail in the Description of the Assessment Procedure section.

The assessments and in-person meetings for decision-making regarding the protocol took place at the Epidemiology and Human Movement (EPIMOV) Laboratory at the Federal University of São Paulo in Santos, Brazil. The target population was the residents in the metropolitan area of Santos, São Paulo, Brazil. The city of Santos is mainly flat and offers a gardened beachfront with an extension of approximately 7 km [22]. Cycle lanes and public equipment widely cover the city to practice PA. It is common to watch people exercising in different spaces of the city. Moreover, it is ranked 5th in Brazil in terms of quality of life [22].

### Randomization Procedures

The randomization sequence (initial randomization and rerandomization) was generated in blocks of 6 participants, using a website [23], and was sealed in opaque envelopes numbered in the opening sequence. The envelopes were prepared by a third person who was not involved in any other study phase. The first envelope was opened at the time of enrollment after participants watched the animated video about the benefits of practicing PA. The second envelope, containing the new group allocation, was kept sealed until the sixth week of the intervention. We opened the second envelope only among nonresponder participants in their presence. After the onset of the COVID-19 pandemic and the suspension of in-person activities at the university, a research team member opened the envelopes and informed the nonresponder participants about the new group intervention, using the same text messaging app.

### Description of Interventions

The adaptive intervention protocol was designed to present different stimuli in a stepwise manner, which could contribute to optimizing the cost-effectiveness of interventions delivered in health services. Based on the ecological model proposed by Bauman et al [4] and Sallis et al [5], the intervention was initially focused on individual (demographic and psychological) and social (social support) factors. By the end of the SMART protocol, it would be possible to analyze if there is an ideal sequence of stimuli to deliver, and thus, strategies in public health could be planned in a more cost-effective sequence, starting with those less demanding in financial and human resources and progressing to those more demanding in financial and human resources.

**Figure 1.**
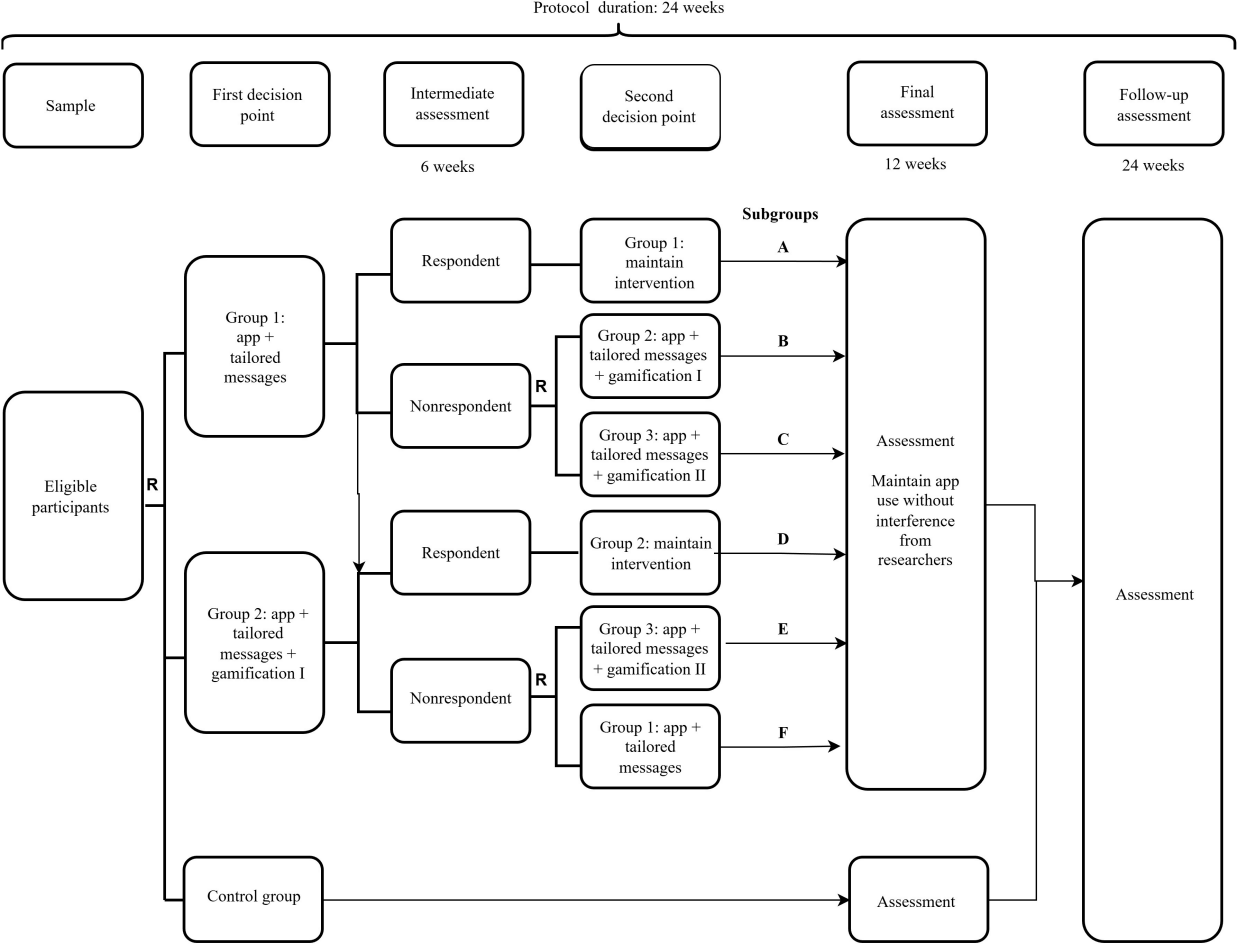
Flowchart of the study protocol. R: randomization.

The intervention lasted a total of 24 weeks and was delivered in 2 phases. Initially, we randomized participants into 3 groups (group 1: app plus tailored messages; group 2: app plus tailored messages plus gamification; and control group: advice) (Figure 1). The first intervention phase lasted 12 weeks, during which the average number of participants’ daily steps was registered weekly in groups 1 and 2. By the sixth week of the intervention, we assessed participants’ performance in terms of daily steps and classified them into responders and nonresponders. We then rerandomized nonresponders, adding a third group (group 3: app plus tailored messages plus gamification II) (Figure 1). After completing 12 weeks, we advised participants to continue using the app and monitor daily steps for over 12 weeks. From that moment on, they did not receive any direct intervention from the researchers. We invited participants from all groups for reassessments at weeks 12 and 24.

Initially, all participants watched an animated video about the benefits of practicing PA and received a printed booklet with the same content as the video. Moreover, we sent them a link so that they could watch the video again as often as they wanted. After this procedure, we opened the first randomization envelope containing the initial group assignment and explained the detailed intervention according to the group allocation.

A detailed description of the intervention has been published elsewhere [20]. Briefly, the approaches used in each group were as follows:

- Group 1: Participants used the app to self-monitor their daily steps. We sent tailored messages weekly using a text messaging app, according to their performance in the last week, and motivated them to increase their step count during the following week.
- Group 2: In addition to the self-monitoring and tailored messages, we instructed participants on how to access the gamification features the app offers, such as ranking, virtual challenges, virtual badges, and social media interaction.
- Control group: We advised participants to increase their PA levels, based on the information provided by the animated video and printed booklet. We did not inform them about using apps. Moreover, since these participants would not be rerandomized, we did not invite them to the intermediate assessment at week 6.
- Group 3: We included the second gamification phase at the intermediate assessment (week 6). Gamification II offered the same features as gamification I plus opportunities for socialization among participants and the research team. We planned in-person meetings every 2 weeks to practice PA in groups and created a group in a text messaging app to facilitate interaction.

Before beginning the study, researchers from the EPIMOV team used smartphone apps to track PA. After a period of use and comparing the features, easiness, and difficulties of the apps, they decided that Pacer (Pacer Health Inc) was the most suitable to use in the trial. It registers the step count and offers behavior change techniques (self-monitoring, goal setting, progress bars, competition, ranking, virtual badges, and social interaction). In addition, we tested the feasibility of the SMART design using Pacer [17].

We used the free version of the Pacer app in this study, providing the behavior change techniques according to group allocation. The participants downloaded the app on their phones with the aid of researchers. In addition to in-person instructions on using the app, we provided a printed booklet with the same instructions. In addition, at any time, participants could contact researchers to ask for help regarding the use of the app. There were no changes to the protocol after the trial commenced, including no changes in the version of the app.

The content of the weekly tailored text messages was developed by one of the researchers supervised by a sports psychologist, according to the stage of behavior change, and is available elsewhere [17]. We created a sequence of messages for precontemplation and contemplation, and another sequence for participants in preparation, action, and maintenance. For each sequence, we had messages for occasions when participants achieved or overcame their goals and messages for occasions when they did not reach their goals. Other than the tailored messages, participants did not receive reminders or prompts to use the app.

The opportunities for socialization offered to participants reallocated to group 3 occurred biweekly as in-person meetings. During these meetings, we practiced PA in groups (participants and researchers), which was planned and conducted by an experienced professional in physical education. All meetings took place on Saturdays at the beachfront, and at the end of the PA sessions, researchers remained available to interact with participants. The sessions lasted, on average, 1 hour and 30 minutes (exercise and postexercise interaction) and included a warm-up period (around 5 minutes) and planned walking or running exercises targeted (each session) at step count, activity duration, or pace. In addition, participants had the option to join a group in the text messaging app so that they could interact with each other and plan the PA meetings.

### Goal Setting and Intermediate Assessment

We set individualized goals for each participant. We instructed them to maintain their routines during the first week of app use. Then, we registered the average number of daily steps collected by the app, and this step count was considered as the participants’ goal. The goal-setting process adopted in this study has been published in another study [20]. Finally, we sent a text message informing participants that their goal was to overcome the initial step count from that day on.

We used the average number of daily steps collected by the app to determine responders and nonresponders at the intermediate assessment. We registered the weekly performance of participants and plotted it in individualized sheets. Then, we used linear regression lines along the performance data during the first 6 weeks of the intervention. Finally, responders were those participants with a positive slope on the regression line, while nonresponders were those with a zero or negative slope on the regression line. We set this assessment to consider the individualized performance of participants over time, attempting to set feasible goals for our sample. If we faced errors when extracting the step count from the app interface, such as a lack of performance updates or operational errors, participants remained in the same group for initial allocation.

After the intermediate assessment, we invited participants for an in-person meeting at the laboratory. In this meeting, we explained their performance during the first 6 weeks of the intervention, and nonresponders were rerandomized (we opened the second sealed envelope that remained stored since the first randomization). Moreover, we provided information about the different components of the intervention (Figure 1). After the onset of the COVID-19 pandemic and the restrictive measures, we conducted the intermediate assessment remotely (one of the researchers opened the second envelope and communicated with participants via text messages).

### Study Measurements

We assessed participants at the beginning of the protocol (initial assessment) and reassessed them at weeks 12 (final assessment) and 24 (follow-up assessment). All of the assessors were blinded regarding the group allocation. The assessment protocol included demographic and socioeconomic information, general health condition, stages of behavior change for PA, level of PA, and anthropometric measures.

The assessors completed the protocol on 2 different days, 7 days apart. On the first day, participants collected their demographic and socioeconomic information, stages of behavior change for PA, and general health status. At the end of day 1, they received an accelerometer to wear during the subsequent 7 days when they returned to complete the assessments. On day 2, participants returned the accelerometer, and we assessed their level of PA and anthropometric measures.

By week 12 (the end of the intervention protocol), we reassessed participants and advised them to keep using the app to track their daily steps, but from this moment on, they did not receive tailored messages or any other direct intervention from the researchers. Finally, at week 24, we invited participants to return for the follow-up assessment, irrespective of whether they had presented for the 12-week evaluation.

At each assessment point, we presented the results of the previous evaluation to participants. At week 12, they received the results from the initial assessment, and at week 24, they received the results from the final evaluation. After the study ended at week 24, the results were provided according to their wish and availability. There were no consequences for participants who did not withdraw their results. The results were stored at a safe location and were available to participants at any time.

### Description of the Assessment Procedure

The tests and interviews were standardized and conducted by trained personnel. Moreover, the equipment was periodically checked and calibrated according to the manufacturer’s instructions. We invited all participants to the follow-up assessment regardless of whether they had presented for the final evaluation.

We collected the following demographic and socioeconomic information: age (years), sex, marital status, education, and occupational status. We used the *Critério Brasil* questionnaire to assess socioeconomic status [24]. This questionnaire contains 15 questions, divided into 3 categories: household items, educational level of the family’s chief, and access to services. According to the total score, participants were classified into 6 socioeconomic strata, from A to D, where A represents the highest socioeconomic status and D represents the lowest socioeconomic status.

For assessing general health conditions, we registered participants’ self-reported personal and family health precedents and medication use. Moreover, we registered the following self-reported cardiovascular risk factors: age 45 years or older for men and 55 years or older for women, family history of cardiovascular diseases, diagnosis of arterial blood hypertension, diagnosis of hyperglycemia or diabetes, diagnosis of hypercholesterolemia or dyslipidemia, and smoking status. We complemented the assessment of cardiovascular risk factors with the evaluation of obesity and physical inactivity.

For evaluating the stages of behavior change for PA, we used the Brazilian version of the transtheoretical model [25], which considers that individuals may, according to the circumstances, transit nonlinearly among different behavior change stages [25]. According to the status of the current practice of PA, the willingness to change the behavior, and the fact that PA is essential to health, participants were classified into one of the following stages: precontemplation, contemplation, preparation, and maintenance [25].

For assessing the level of PA, participants received a triaxial accelerometer (Actigraph) at the end of the first day of assessments, which was required to be used for 7 consecutive days and worn over the hip according to the self-reported dominant side of the body. Data were considered valid when available for at least 4 days, including 1 weekend day, and for at least 10 hours per day [26]. When a participant presented with invalid data for the first time, we returned the accelerometer and asked them to use it for more than 7 days. We extracted accelerometer data using the manufacturer’s software and registered the following information: average number of steps per day, time spent in sedentary behavior (0 to 99 step counts/min) [27], and time spent in MVPA (≥1952 step counts/min) [27].

With regard to anthropometric measures, we registered the height (m) and body mass (kg) of participants using a digital scale with a stadiometer (Toledo). We positioned participants standing barefoot over the equipment and asked them to keep looking forward during the measurement. For height measurement, participants kept their arms crossed over the trunk while sustaining maximal inspiration, and for body mass measurement, they kept their arms along the body.

### Sample Size Calculation

Considering a fixed *k* probability, at least *m* participants would be allocated to subgroups B and E, as demonstrated in Figure 1. Moreover, based on a nonresponse rate *q* of 35%‐65% in SMART studies, we estimated that 3 participants per group would be sufficient to ensure familiarity with the protocol, allow identification, and deal with potential difficulties that could occur during the intervention period. Thus, for a probability *k* of 90% and a nonresponse rate *q* of 50% by the end of the sixth week of the protocol, the study should include at least 42 participants [28]. Due to the lack of a similar study to use data for estimating the sample size, after 42 participants completed the protocol, we planned to perform a new sample size calculation and continue the study until the sample size target was achieved. However, this approach was not possible because the protocol had to be interrupted after the onset of the COVID-19 pandemic. Therefore, we completed the study after including 53 participants.

### Data Analysis

Participants’ characteristics are presented using descriptive statistics, such as mean and SD for continuous variables, and absolute number and proportion for categorical variables. We compared the characteristics of participants among groups using 1-way ANOVA for continuous variables and the chi-square test for categorical variables.

We checked for participant performance in groups 1 and 2, using graphs with linear regression lines. For each group and participant, we plotted 3 graphs using the average number of daily steps: 1 with data from the 12 weeks of the intervention, 1 with data from the first 6 weeks (between the first and intermediate assessments), and 1 with data from the last 6 weeks (between the intermediate and final assessments).

To analyze the effects of the intervention on primary and secondary outcomes, we used repeated measures linear mixed models, considering the initial group allocation and time as fixed effects and the individual as a random effect. Moreover, we grouped participants from groups 1 and 2 according to their response to the intervention (responders and nonresponders) and compared their performance using repeated measures linear mixed models, considering response to the intervention and time as fixed effects and the individual as a random effect.

We managed missing data using intention-to-treat analysis by multivariate data imputation, adjusted by sex and age. The graphs for weekly performance were generated using Microsoft Excel (Microsoft Corporation), and data analysis was conducted using Stata version 14 (StataCorp LLC).

## Results

**Figure 2.**
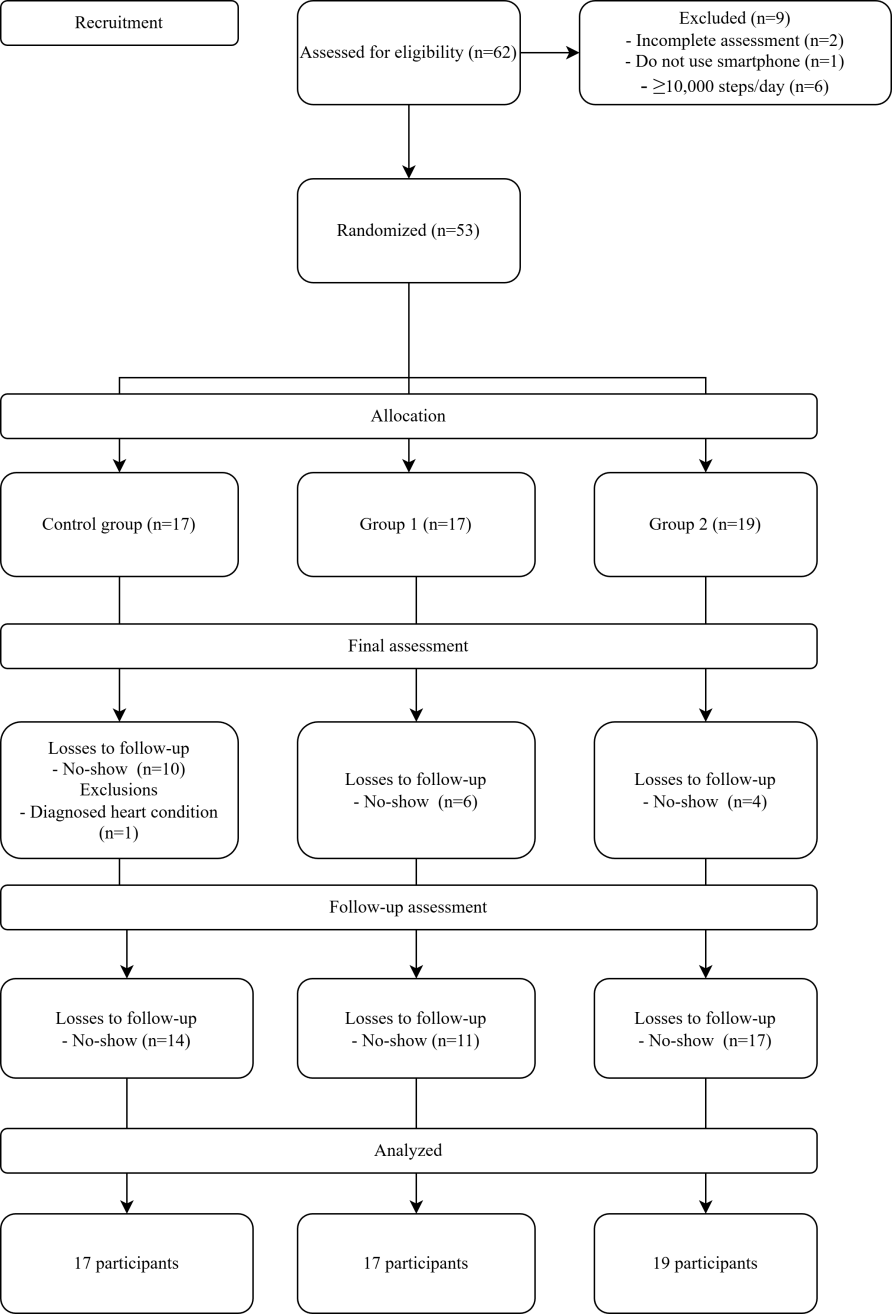
Flowchart of participants in the study.

Of 62 potential individuals who volunteered to participate, 53 met the inclusion criteria and were included in the protocol. Some participants were lost to follow-up, especially at the 24th-week assessment, mainly due to the COVID-19 pandemic. For the same reason, we had to interrupt the protocol and could not achieve a larger sample size. As shown in Figure 2, according to the intention-to-treat analysis, we included all participants in the data analysis.

As we suddenly had to interrupt the inclusion of new participants, there were differences in the number of participants between group 2 and the other groups. Furthermore, our plan to conduct a second trial phase, using intermediate data to estimate sample size, could not be executed.

Protocol deviations were continuously checked, such as accessing app features that were not advised. Only 1 participant from group 1 explored the app and joined its gamification features, such as ranking and challenges of walking distance. There were no harmful events or unintended effects during the study. According to the taxonomy proposed by Michie et al [29], the behavior change techniques in our study included rewards (virtual badges provided by the app), incentives (tailored messages), graded tasks (overcoming the goal), feedback and monitoring (self-monitoring using the app and tailored messages), goals and planning (individual goal setting), social support (practical and general; provided by the app via teaming up with other participants, and for group 3, provided via in-person meetings and text messaging), comparison of behavior (social comparison provided by the ranking in the app), and instructions on how to perform a behavior (provided by the educational material).

In general, participants were middle-aged adults (mean age 44.0, SD 12.7 years), were mainly female (30/53, 57%), and were overweight or obese (mean BMI 29.8, SD 6.5 kg/m^2^). Regarding the stage of behavior change for PA, almost half of the sample (24/53, 45%) was in the preparation stage. As shown in Table 1, there were no differences in participant characteristics, except for sex (*P*=.03) and the stage of behavior change (*P*=.047).

**Table 1.** Characteristics of participants at baseline.

Variable	All participants (n=53)	Control group (n=17)	Group 1 (n=17)	Group 2 (n=19)	*P* value^a^
Age (years), mean (SD)	44.0 (12.7)	45.2 (13.4)	47.7 (11.2)	39.6 (12.5)	.15
Sex (female), n (%)	30 (57)	9 (53)	6 (35)^b^	15 (79)	.03
Body mass (kg), mean (SD)	83.3 (21.9)	89.1 (28.8)	85.1 (14.7)	76.5 (19.3)	.21
Stature (m), mean (SD)	1.66 (0.08)	1.67 (0.08)	1.68 (0.09)	1.65 (0.07)	.49
BMI (kg/m^2^), mean (SD)	29.8 (6.5)	31.5 (8.5)	30.2 (4.7)	28.0 (5.8)	.26
Race, n (%)					.15
White	33 (62)	12 (71)	7 (41)	14 (74)	
Black	6 (11)	0 (0)	4 (24)	2 (11)	
Brown	12 (23)	4 (24)	6 (35)	2 (11)	
Do not know/not reported	2 (4)	1 (6)	0 (0)	1 (5)	
Education, n (%)					.32
Middle school, incomplete	2 (4)	0 (0)	1 (6)	1 (5)	
Middle school, complete	2 (4)	1 (6)	1 (6)	0 (0)	
High school, complete	28 (53)	13 (77)	7 (41)	8 (42)	
Higher education, complete	13 (25)	3 (18)	4 (24)	6 (32)	
Postgraduation, complete	8 (15)	0 (0)	4 (24)	4 (21)	
Employed (yes), n (%)	39 (74)	11 (65)	14 (82)	14 (74)	.62
Socioeconomic classification^c^, n (%)					.20
A	7 (13)	2 (12)	0 (0)	5 (26)	
B1	7 (13)	1 (6)	4 (24)	2 (11)	
B2	18 (34)	5 (29)	6 (35)	7 (37)	
C1	9 (17)	6 (35)	2 (12)	1 (5)	
C2	10 (19)	3 (18)	4 (24)	3 (16)	
D	2 (4)	0 (0)	1 (6)	1 (5)	
Stage of behavior change, n (%)					.047
Contemplation	1 (2)	0 (0)	0 (0)	1 (5)	
Preparation	24 (45)	6 (35)	9 (53)	9 (47)^d^	
Action	13 (25)	4 (24)	1 (6)^b^	8 (42)^d^	
Maintenance	15 (28)	7 (41)	7 (41)^b^	1 (5)^d^	

a *P* values refer to comparisons among groups.

b Significant difference between group 1 and group 2.

c For socioeconomic classification, A represents the highest socioeconomic status and D represents the lowest socioeconomic status.

d Significant difference between the control group and group 2.

All groups were similar when considering the outcome measures. The nonresponse rate was 53% (28/53), without differences in the proportion of respondents at week 6 (*P*=.38) (Table 2).

**Table 2.** Baseline assessment and comparison among groups.

Variable	All participants (N=53)	Control group (n=17)	Group 1 (n=17)	Group 2 (n=19)	*P* value^a^
Average daily step count, mean (SD)	6134 (1932)	6616 (2069)	5651 (1810)	6170 (1942)	.44
Average time spent in moderate-to-vigorous physical activity (min/day), mean (SD)	33 (16)	39 (14)	27 (18)	33 (13)	.17
Average time spent in sedentary behavior (min/day), mean (SD)	671 (130)	669 (77)	678 (154)	664 (150)	.96
Respondent at week 6 (yes), n (%)	—^b^	—	10^c^ (63)	7^d^ (47)	.38
Group allocation after rerandomization, n (%)					.003
Group 1	—	0 (0)	14 (82)	5 (26)	
Group 2	—	0 (0)	3 (18)	10 (53)	
Group 3	—	0 (0)	0 (0)	4 (21)	

a *P* values refer to comparisons among groups.

b Not applicable.

c Out of a total of 16.

d Out of a total of 15.

Table 3 presents the outcome measures in each group at each assessment (initial, final, and follow-up). We managed missing data using multivariate multiple imputation adjusted for sex and age.

**Table 3.** Effects of the intervention within and between groups.

Variable	Control group^a^	Group 1^a^	Group 2^a^
Average daily step count, mean (SD)			
Initial (baseline)	6606 (1989)^b^	5643 (1740)^b^^,c^	6180 (1887)^c^
Final (week 12)	7063 (2215)	7617 (1873)^d^	7524 (2256)^d^
Follow-up (week 24)	6271 (1574)	8480 (2387)	6350 (2413)
Average time spent in moderate-to-vigorous physical activity (min/day), mean (SD)			
Initial (baseline)	39 (14)^c^	27 (18)^b^^,e^	33 (13)
Final (week 12)	36 (20)^d^	35 (21)^d^	36 (14)
Follow-up (week 24)	41 (19)	44 (15)	33 (17)
Average time spent in sedentary behavior (min/day), mean (SD)			
Initial (baseline)	670 (74)^c^	678 (148)^c^	663 (144)^c^
Final (week 12)	569 (129)^d^	644 (137)^d^	613 (102)^d^
Follow-up (week 24)	694 (137)	677 (117)	663 (132)

a Missing data were treated using multivariate multiple imputation adjusted by sex and age.

b Significant difference within groups (initial vs follow-up assessment).

c Significant difference within groups (initial vs final assessment).

d Significant difference within groups (final vs follow-up assessment).

e Significant difference between groups (control group vs group 1).

For the primary outcome (the average count of daily steps), we observed differences in the factor *time* and at the individual level (Table 4). While participants from the control group showed a reduction in the step count over time (follow-up vs initial assessment: β=−575.8; *P*<.05) and participants from group 2 showed an increase in the step count between the initial and final assessments (β=897.4; *P*<.001), participants from group 1 showed an increase in the step count for all assessments (final vs initial assessment: β=650.2; *P*<.001; follow-up vs initial assessment: β=1521.9; *P*<.001).

**Table 4.** Average daily step count considering group, time, and group and time.

Variable^a^	Coefficient, value (95% CI)	*P* value
Group (Ref^b^: control group)		
Group 1	−895.2 (−2179.3 to 388.8)	.17
Group 2	−903.2 (−2158.7 to 352.2)	.16
Time (Ref: I^c^)		
Fi^d^	−147.0 (−380.2 to 86.1)	.22
Fo^e^	−575.8 (−1045.3 to −106.2)	.02
Group 1 and time (Ref: control group and I)		
Fi	797.2 (475.3 to 1119.1)	<.001
Fo	2097.6 (1577.2 to 2618.1)	<.001
Group 2 and time (Ref: control group and I)		
Fi	1044.4 (747.0 to 1341.8)	<.001
Fo	982.8 (332.9 to 1632.6)	<.001
Contrasts, control group		
Fi and I	−147.0 (−380.2 to 86.1)	.22
Fo and I	−575.8 (−1045.3 to −106.2)	.02
Fo and Fi	−428.7 (−920.3 to 63.1)	.09
Contrasts, group 1		
Fi and I	650.2 (428.2 to 872.1)	<.001
Fo and I	1521.9 (1297.4 to 1746.4)	<.001
Fo and Fi	871.7 (640.4 to 1103.0)	<.001
Contrasts, group 2		
Fi and I	897.4 (712.8 to 1082.0)	<.001
Fo and I	407.0 (−42.2 to 856.2)	.08
Fo and Fi	−490.3 (−949.3 to −31.4)	.04

a Contrasts within groups over time (linear mixed model analysis). Fixed effects: initial group allocation and time of assessment; random effect: participant.

b Ref: reference.

c I: initial assessment.

d Fi: final assessment.

e Fo: follow-up assessment.

Results from the analysis of the time spent in sedentary behavior are presented in Table 5. We observed differences in the factor *time* in all groups (final vs initial assessment: β=−70.8; *P*<.001) and at the individual level (*P*<.001), without differences among groups.

**Table 5.** Time spent in sedentary behavior considering group, time, and group and time.

Variable^a^	Coefficient, value (95% CI)	*P* value
Group (Ref^b^: control group)		
Group 1	−6.9 (−81.9 to 68.1)	.86
Group 2	−20.3 (−93.8 to 53.3)	.59
Time (Ref: I^c^)		
Fi^d^	−70.8 (−88.8 to −52.9)	<.001
Fo^e^	19.7 (−16.4 to 55.9)	.29
Group 1 and time (Ref: control group and I)		
Fi	26.0 (1.2 to 50.7)	.04
Fo	−23.8 (−63.9 to 16.2)	.24
Group 2 and time (Ref: control group and I)		
Fi	41.9 (19.0 to 64.8)	<.001
Fo	−10.3 (−60.3 to 39.7)	.69
Contrasts, control group and time		
Fi and I	−70.8 (−88.8 to −52.9)	<.001
Fo and I	19.7 (−16.4 to 55.9)	.29
Fo and Fi	90.5 (−88.8 to −52.9)	<.001
Contrasts, group 1 and time		
Fi and I	−44.9 (−61.9 to −27.8)	<.001
Fo and I	−4.1 (−21.4 to 13.2)	.64
Fo and Fi		
Contrasts, group 2 and time		
Fi and I	−29.0 (−43.2 to −14.7)	<.001
Fo and I	9.4 (−25.2 to 43.9)	.59
Fo and Fi	38.3 (3.04 to 73.6)	.03

a Contrasts within groups over time (linear mixed model analysis). Fixed effects: initial group allocation and time of assessment; random effect: participant.

b Ref: reference.

c I: initial assessment.

d Fi: final assessment.

e Fo: follow-up assessment.

We also observed differences at the individual level for the time spent in MVPA (Table 6). For the factors *group* and *time*, participants from the control group showed a reduction in the time spent in MVPA from the initial assessment to the final assessment (β=−7.9; *P*<.001) and then showed an increase in the time by the follow-up assessment (β=9.0; *P*<.001). On the other hand, participants from group 1 showed an increase in the time spent in MVPA at the follow-up assessment (β=7.8; *P*<.001).

**Table 6.** Time spent in moderate-to-vigorous physical activity considering group, time, and group and time.

Variable^a^	Coefficient, value (95% CI)	*P* value
Group (Ref^b^: control group)		
Group 1	−13.0 (−22.4 to −3.5)	.007
Group 2	−5.5 (−14.8 to 3.8)	.24
Time (Ref: I^c^)		
Fi^d^	−7.9 (−9.9 to −5.9)	<.001
Fo^e^	1.1 (−2.9 to 5.2)	.59
Group 1 and time (Ref: control group and I)		
Fi	9.2 (6.4 to 11.9)	<.001
Fo	7.9 (3.4 to 12.4)	.001
Group 2 and time (Ref: control group and I)		
Fi	8.8 (6.2 to 11.4)	<.001
Fo	−2.6 (−8.2 to 3.0)	.37
Contrasts, control group and time		
Fi and I	−7.9 (−9.9 to −5.9)	<.001
Fo and I	1.1 (−2.9 to 5.2)	.59
Fo and Fi	9.0 (4.8 to 13.3)	<.001
Contrasts, group 1 and time		
Fi and I	1.2 (−0.7 to 3.2)	.20
Fo and I	9.1 (7.1 to 11.0)	<.001
Fo and Fi	7.8 (5.8 to 9.8)	<.001
Contrasts, group 2 and time		
Fi and I	0.9 (−0.7 to 2.5)	.27
Fo and I	−1.5 (−5.4 to 2.4)	.46
Fo and Fi	−2.4 (−6.3 to 1.6)	.25

a Contrasts within groups over time (linear mixed model analysis). Fixed effects: initial group allocation and time of assessment; random effect: participant.

b Ref: reference.

c I: initial assessment.

d Fi: final assessment.

e Fo: follow-up assessment.

**Figure 3.**
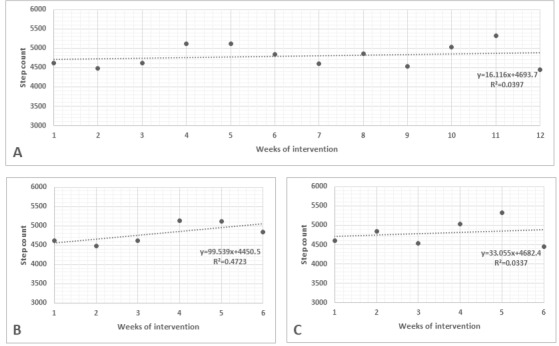
Weekly performance of participants from group 1. (A) Performance from week 1 to week 12; (B) performance from week 1 to week 6 (before rerandomization); (C) performance from week 7 to week 12 (after rerandomization).

Figures3  4 present the performance of participants from groups 1 and 2 during the 12 weeks of the protocol and from weeks 1 to 6 and weeks 7 to 12 (before and after rerandomization, respectively).

**Figure 4.**
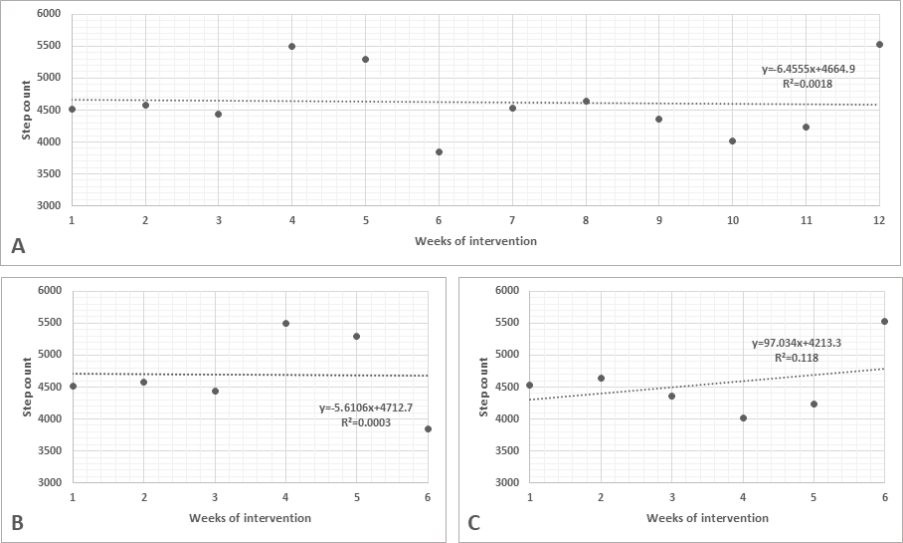
Weekly performance of participants from group 2. (A) Performance from week 1 to week 12; (B) performance from week 1 to week 6 (before rerandomization); (C) performance from week 7 to week 12 (after rerandomization).

In Table 7, we present the analysis of the average daily step count of responders and nonresponders, regardless of group allocation. Using the Pacer app, we collected these data in weeks 1, 6, and 12. We observed differences at the individual level, and although all participants showed reductions in the average daily step count over time (week 6 vs week 1: β=−496.7; *P*<.001; week 12 vs week 1: β=−579.3; *P*<.001), among responders, the step count constantly increased (week 6 vs week 1: β=2044.7; *P*<.001; week 12 vs week 1: β=2299.6; *P*<.001).

**Table 7.** Pacer app’s average daily step count from responders and nonresponders, regardless of group allocation, according to the response to the intervention and time.

Variable^a^	Coefficient, value (95% CI)	*P* value
Response to the intervention (Ref^b^: no)		
Yes	−1588.8 (−3252.4 to 74.9)	.06
Time (Ref: week 1)		
Week 6	−496.7 (−651.6 to −341.8)	<.001
Week 12	−579.3 (−743.6 to −414.9)	<.001
Response and time (Ref: no and week 1)		
Yes and week 6	2044.7 (1835.5 to 2253.9)	<.001
Yes and week 12	2299.6 (2076.1 to 2523.0)	<.001
Contrasts, nonresponders and time		
Week 6 and week 1	−496.7 (−651.6 to −341.8)	<.001
Week 12 and week 1	−579.3 (−743.6 to −414.9)	<.001
Week 12 and week 6	−82.6 (−246.9 to 81.7)	.33
Contrasts, responders and time		
Week 6 and week 1	1548.0 (1407.4 to 1688.6)	<.001
Week 12 and week 1	1720.3 (1568.8 to 1871.7)	<.001
Week 12 and week 6	172.3 (20.8 to 323.8)	.03

a Contrasts between response and time (linear mixed model analysis). Fixed effects: response to the intervention and time of assessment; random effect: participant.

b Ref: reference.

## Discussion

This study aimed to investigate the effects of using a smartphone app combined with behavior change techniques on the level of PA among adults and older adults. Due to the sudden interruption of the protocol after the onset of the COVID-19 pandemic, we completed the study with a small sample size. Overall, all participants presented with improvements in outcomes over time. Specifically for PA, all participants from group 1 showed a consistent increase in the average daily step count. According to the benchmarks for assessing the effectiveness of interventions to promote PA proposed by Wright et al [30], considering the digital intervention setting, participants from the control group presented a performance below the 25th percentile for step count, while participants from groups 1 and 2 performed greater than the 75th percentile and between the 50th and 75th percentiles, respectively.

Only participants from group 1 continued to increase the step count even after the end of the intervention. Despite the nonstatistical difference in the proportion of respondents and nonrespondents between groups, which may have occurred due to the small sample size and consequently low statistical power, it is possible that the stepwise offering of intervention components (initiation with less complex strategies and gradual increases according to participants’ responses) may be more effective in supporting behavior change than offering more complicated strategies. Furthermore, gamification may be more effective for specific individual profiles and may not be as attractive to others. We support this hypothesis because participants from group 1 had positive regression lines over the 12 weeks of the intervention.

Interestingly, the time spent in sedentary behavior and the time spent in MVPA did not present the same trajectory as step count. The increase in the average daily step count was not accompanied by a reduction in the time spent in sedentary behavior or an increase in the time spent in MVPA, which can be explained by the outcome we measured. Participants may have altered their time for physical activities that we did not assess. Although the step count and the time spent in sedentary behavior are related [21], a systematic review and meta-analysis also found increases in step count not accompanied by reductions in sedentary behavior [31].

Our results are similar to those of Vandelanotte et al [32], who did not observe differences in the level of PA among 3 groups (1 control and 2 intervention groups). Despite differences in the study design and intervention protocol between the studies, both similarly associated the use of technology with behavior change techniques. The clinical trial conducted by Plotnikoff et al [33] found benefits in PA and general health among participants with type 2 diabetes, presenting a considerably higher effect size (Cohen *d*=0.67 for step count). Their protocol associated using a smartphone app with in-person meetings for group therapy and outdoor physical exercises [33]. It may be possible that, beyond the regular in-person meetings, a population diagnosed with chronic conditions, such as diabetes, may be more motivated to become more active.

Likewise, other clinical trials aiming to increase the level of PA found better results in populations with clinical conditions than studies involving a healthy population [31]. In a systematic review and meta-analysis, Ringeval et al [31] showed that almost 60% of clinical trials that used an electronic device to monitor the level of PA included individuals at risk or already presenting with a clinical condition, and only 1 study included healthy individuals as the target population.

The nonresponse rate observed in our study (28/53, 53%) was lower than the expected rate (65%). This information may contribute to estimating the nonresponse rate in the planning of future clinical trials aiming to increase the level of PA in adaptive intervention designs, as well as the planning of adaptive intervention protocols with more decision points for rerandomization and changing the stimuli to favor behavior change in the practice of PA.

Respondents in week 6, independently of the initial or intermediate randomized allocation, constantly increased their average daily step count, while nonrespondents showed negative performance. Since our protocol did not include more decision points, it is impossible to know if these participants would benefit from more rerandomizations. These findings led us to reflect on the response at the intermediate assessment, which could be a prognostic indicator of the practice of PA. In this sense, Shang et al [34] pointed out the importance of deepening the investigation of fluctuating behavior in PA.

It is important for future studies to investigate if nonrespondents at the first phase of the intervention would benefit from new stimuli. In addition, a study with a larger sample size could investigate if nonrespondents present characteristics that could help health professionals and services to identify the population at higher risk for physical inactivity and thus design more personal approaches [34]. When planning interventions to increase PA, planning beyond the activity itself is necessary. As mentioned earlier, psychosocial, economic, and environmental factors are involved in this challenge [4]. Furthermore, there is a need to consider the dynamic characteristics of behavior change, which may include relapse periods and difficulties in maintaining the new behavior [34,35].

The loss to follow-up in our study, except for the control group, was consistent with the expectation for an interventional study to increase PA, which varies from 20% to 35% [32,33]. We faced difficulties following participants from the control group even before the onset of the COVID-19 pandemic. Our hypotheses for these losses are as follows: (1) it is possible that researchers and participants from groups 1 and 2 developed a bond due to the weekly text message interaction, which may have contributed to keeping the appointments for reassessments, and (2) it is possible that some of the participants may have volunteered for our study with the expectation of using an app for PA, which was not met for participants from the control group. For future studies, as a strategy to increase adherence among control participants, it may be helpful to offer an app without associating other behavior change techniques or even contact them periodically to develop a bond.

Many individuals have difficulty establishing effective strategies to change their behavior regarding PA. As pointed out by Warburton and Bredin [36], there is enough knowledge about the effects and benefits of the practice of PA, and the current challenge lies in translating knowledge into strategies to increase PA at a populational level. In our study, we tried to encompass different determinants of PA [4,5] at the individual level (eg, text messages and self-monitoring could support motivation) and the interpersonal level (eg, social support from the research team, in-person activities in group 3, and establishment of cultural norms among participants); provide a social or cultural environment through app features, such as ranking and groups/teams; and build an information environment through counseling and education.

After analyzing the performance of participants in this study, we suggest that intervention protocols aiming to increase the level of PA in research, clinical, and collective contexts should consider the adaptive intervention design as a viable and potentially effective alternative, as well as the association with environmental and institutional interventions [1]. The recommendations of the World Health Organization Global Action Plan [3] and ecological models [4,5] reinforce the complexity of this behavior change.

Finally, it is essential to highlight that recommendations about the ideal quantity of PA are being constantly revised. Considering the current recommendation of the World Health Organization for the adult population that PA of any amount and intensity is better than no PA [37] and the findings from Warburton and Bredin [36] that there are benefits of transitioning from a lower to a higher level of PA independently of the amount, we believe that our protocol was effective, as all participants presented some increase in the average daily step count.

As strengths of our study, we highlight the adaptive intervention design, which is still not explored in PA, and its association with behavior change techniques and complex technology. Moreover, despite the small sample size, we found relevant information that may contribute to advancing knowledge for PA promotion.

Our study had some limitations. Although we completed the first phase of the study, the second phase was not possible due to the onset of the COVID-19 pandemic. Data analysis was limited due to the small sample size and losses to follow-up, especially after the onset of the pandemic, which led to the sudden interruption of the protocol. Although we managed this limitation using adequate statistical treatment of the data, the analysis may be underpowered. Another limitation regarding the data analysis was the intention-to-treat approach adopted in our study, which could underestimate the impact of other factors influencing participation in the study and adherence to the protocol [38]. Finally, the characteristics of the sample, such as a higher level of education and having access to technologies, may limit the generalization of the results.

As observed in other PA studies, most participants completed at least high school [32,39,40]. Epidemiological studies have indicated that a higher education level and income are associated with higher practice of leisure-time PA [4]. One of the challenges in designing programs for PA is engaging individuals who are less likely to participate in leisure-time PA due to the perception of limited time, a limited understanding of the effects of the practice of PA, or fatigue resulting from physically demanding work [40]. The socioeconomic characteristics of our sample may have contributed to our results.

In conclusion, our adaptive intervention protocol using a smartphone app combined with behavior change techniques led to increases in the level of PA over time, especially among participants from group 1. Participants from group 1 showed increases in PA levels at all assessments, while those from group 2 showed increases only at the final assessment when compared with the initial assessment. Moreover, there was a time effect for the increase in the time spent in MVPA. It is possible that offering stepwise new stimuli and behavior change techniques may contribute positively to the process of behavior change regarding PA. We acknowledge that the small sample size may limit the interpretation of the results.

## Supplementary material

10.2196/73388 Checklist 1 CONSORT‐EHEALTH checklist (V 1.6.1).

## Abbreviations

CONSORT: Consolidated Standards of Reporting Trials
EPIMOV: Epidemiology and Human Movement
MVPA: moderate-to-vigorous physical activity
PA: physical activity
SMART: sequential multiple assignment randomized trial
